# Real-world applicability of steatotic liver disease nomenclature: multifactorial phenotypes as an unmet classification need

**DOI:** 10.1007/s11739-026-04373-3

**Published:** 2026-05-07

**Authors:** Rosanna Villani, Elisabetta Cornacchia, Gaetano Serviddio

**Affiliations:** 1 Liver Unit, C.U.R.E (University Center for Liver Disease Research and Treatment), University Hospital Policlinico Foggia, Foggia, Italy; 2 Department of Medical and Surgical Sciences, University of Foggia, Foggia, Italy

**Keywords:** MASLD, Steatotic liver disease, SLD classification, MetALD

Dear Editor,

The multisociety Delphi consensus statement published in 2023 by Rinella et al. introduced a revised nomenclature for hepatic steatosis, proposing steatotic liver disease (SLD) as an umbrella definition encompassing metabolic dysfunction-associated steatotic liver disease (MASLD), metabolic and alcohol-associated liver disease (MetALD), and secondary hepatic steatosis [1]. This consensus represents a milestone in hepatology, providing a clinically oriented classification for the most prevalent chronic liver disease worldwide [2, 3]. The Rinella 2023 guidelines explicitly state that multiple etiologies of steatosis can coexist and that when additional drivers of steatosis are identified, all of them should be considered [1]. However, the current nomenclature acknowledges the phenomenon but does not provide operational classification categories for combined SLD other than MetALD. Therefore, it remains unclear whether the 2023 framework captures the full spectrum of SLD presentations encountered in clinical practice. This issue derives from a clinical observation: in real-world practice, a substantial proportion of patients with SLD present with more than one concurrent etiologic driver, but no guideline-defined category exists for defining this subgroup of patients.

We retrospectively applied the Rinella 2023 nomenclature to 1414 consecutive adult patients referred to our Liver Unit for SLD assessment between January 2023 and June 2025. SLD was diagnosed by abdominal ultrasonography and confirmed by controlled attenuation parameter (CAP) measurement. Each patient was systematically assessed for metabolic criteria, alcohol consumption, and secondary causes of hepatic steatosis including chronic HCV infection, endocrine disorders, drugs, genetic diseases, or toxic exposures according to the criteria proposed by Rinella et al. [1] and Liebe et al. [4]. Endocrine evaluation included TSH, fasting insulin, and hormonal profiling as clinically indicated; drug history was collected using a structured questionnaire covering all agents known to cause secondary hepatic steatosis; genetic causes were investigated in selected cases based on clinical suspicion. For the purposes of this analysis, patients meeting criteria for a single guideline-based category were defined according to current nomenclature as MASLD, MetALD, ALD or secondary SLD, and those whose phenotype exceeded the current framework as multifactorial. The study was approved by the local Ethics Committee (No. 58/CE/2025).

A total of 1,414 patients were included (60.4% male; median age 59.9 years, IQR 17). Using the Rinella 2023 classification, 1094 patients (77.4%) were correctly classified within a guideline-defined category: 822 patients (58.1%) had MASLD, 242 (17.1%) had MetALD, 25 (1.8%) had ALD, and 5 (0.4%) had secondary SLD. Overall, 320 patients (22.6%) exceeded the current classification framework: 254 (18.0%) had MASLD in association with at least one secondary cause of steatosis, 2 (0.1%) had ALD combined with a secondary cause, and 64 (4.5%) presented with complex SLD involving multiple potentially etiologic drivers (see Table 1).

**Table 1:**
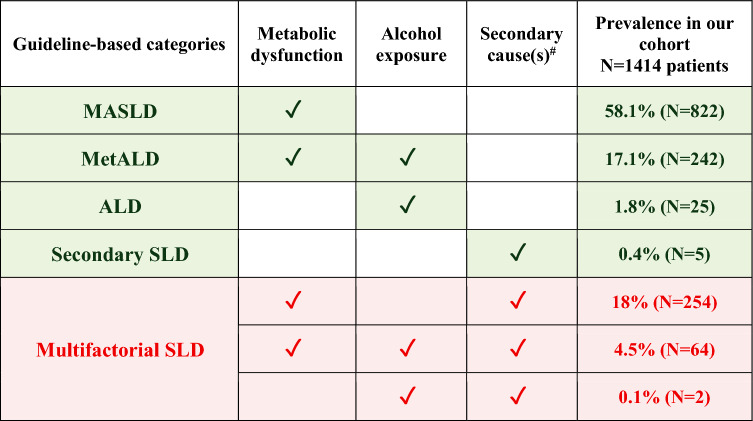
Steatotic liver disease phenotypes and their classification status according to current guidelines

Phenotype without a corresponding guideline-defined category is highlighted in red

Secondary causes include chronic HCV infection, endocrine disorders (hypothyroidism, GH deficiency, PCOS), drugs (amiodarone, corticosteroids, methotrexate, valproic acid, tamoxifen and others), genetic diseases (LALD, Wilson disease, hypobetalipoproteinemia, glycogen storage diseases), nutritional-related causes, and chronic exposure to environmental toxins (according to the full list provided by Liebe et al. [4])

*SLD* steatotic liver disease, *ALD* alcohol-associated liver disease, *MASLD* metabolic dysfunction–associated steatotic liver disease, *MetALD* MASLD + ALD

^**#**^at least one secondary cause

Among the 1382 patients meeting MASLD criteria, 822 (59.5%) had MASLD alone whereas 560 (40.5%) had MASLD with at least one additional etiologic driver.

These findings suggest that nearly one in four patients referred for SLD evaluation cannot be fully classified within a single guideline-defined category. The most numerically relevant unclassifiable group was MASLD associated with at least one secondary cause of steatosis (18%), a clinically plausible and likely under-recognized scenario given the high prevalence of metabolic dysfunction and concurrent secondary conditions such as hypothyroidism or drug-induced steatosis. Importantly, not all secondary drivers could carry the same clinical weight: transient hypothyroidism might contribute minimally, whereas long-term tamoxifen therapy in women with MASLD, whose hepatotoxic effect is well-documented [5, 6], may represent an additive and clinically meaningful etiologic burden. The relative weight of each secondary driver in contributing to hepatic steatosis and, eventually, liver injury remains poorly defined and warrants further dedicated investigation. These observations suggest that systematic evaluation of all potential etiologic drivers should be considered in the clinical work-up of SLD patients.

Moreover, the etiologic profile of SLD patients may change over time, highlighting the need to reassess all potential drivers at each clinical follow-up. A patient initially meeting criteria for MASLD may subsequently have additional etiologic contributors, such as a new drug exposure or an emerging endocrine disorder, thus shifting toward a multifactorial phenotype.

Our data suggest that the lack of a systematic assessment of all potential etiologic drivers may affect patient selection in observational studies and clinical trials using guideline-defined SLD categories as inclusion criteria, since unrecognized multifactorial phenotypes may introduce heterogeneity and misclassification bias. This challenge has also been discussed by Baffy et al. [7] and Israelsen et al. [8], who addressed unresolved classification issues within the current framework, including the arbitrary alcohol thresholds to distinguish MetALD from ALD, and the highly dynamic nature of SLD subclassification, with direct implications for clinical trial eligibility and patient management. Our data extend this concept by showing that a similar gap can be observed in patients with coexisting metabolic and secondary etiologic drivers.

Some limitations of our study should be considered. The study was conducted in a single tertiary center, which may overestimate the prevalence of complex phenotypes compared to primary care settings. Moreover, the retrospective analysis of secondary causes may have led to underestimation of some drivers.

In conclusion, the application of the Rinella 2023 nomenclature to a real-world cohort of 1414 patients reveals that 22.6% of individuals presenting with SLD cannot be classified within a single guideline-defined category. These findings underscore the importance of systematically evaluating all potential etiologic drivers in every patient with SLD, not only at the initial assessment but also at each clinical follow-up, as the etiologic profile may change over time. Recognizing multifactorial phenotypes has direct implications for patient characterization, clinical decision-making, and the design of future epidemiological and interventional research.

## Data Availability

The data supporting the findings of this study are available within the article.

## Declarations

### AI assistance

The authors did not use AI for the drafting of this manuscript.
